# A three-month longitudinal study of changes in day/night serum total antioxidant capacity in paranoid schizophrenia

**DOI:** 10.1371/journal.pone.0189348

**Published:** 2017-12-08

**Authors:** Armando L. Morera-Fumero, Estefanía Díaz-Mesa, Pedro Abreu-Gonzalez, Lourdes Fernandez-Lopez, Fernando Guillen-Pino

**Affiliations:** 1 Departamento de Medicina Interna, Dermatología y Psiquiatría, Facultad de Ciencias de la Salud, Universidad de la Laguna (ULL), La Laguna, Santa Cruz de Tenerife, España; 2 Consultoría Psiquiátrica SC, Santa Cruz de Tenerife, España; 3 Servicio de Psiquiatría, Hospital Universitario de Canarias, La Laguna, Santa Cruz de Tenerife, España; 4 Departamento de Ciencias Médicas Básicas: Unidad de Fisiología, Facultad de Ciencias de la Salud, Universidad de la Laguna (ULL), La Laguna, Santa Cruz de Tenerife, España; 5 Sociedad para la Investigación y Asistencia en Salud Mental, Santa Cruz de Tenerife, España; Peking University, Institute of Mental Health, CHINA

## Abstract

Free radicals and an oxidant/antioxidant imbalance have been involved in the schizophrenia pathophysiology. The total antioxidant capacity (TAC) is a measure of the antioxidant capacity of a system. Day/night changes are a biological characteristic of hormones such as melatonin or cortisol. There is little information about TAC day/night changes in schizophrenia patients. The aim of this research is to study if there are day/night changes in serum TAC levels of schizophrenia patients. Thirty-two DSM-IV schizophrenia paranoid patients were studied. Blood was sampled at 12:00 and 00:00 h at admission, discharge and three months after hospital discharge (TMAHD). TAC results are expressed as mmol of Trolox/L. Patients did not have day/night TAC differences at admission (12:00: 0.67±0.12 vs. 00:00: 0.61±0.14, p>0.14) or discharge (12:00: 0.65±0.15 vs. 00:00: 0.65±0.12, p>0.99). At TMHD, patients had significantly higher TAC levels at midday than midnight (12:00: 0.83±0.10 vs. 00:00: 0.74±0.12, p<0.006) as it has been reported in healthy subjects. There were no significant TAC differences at 12.00 and 00:00 between admission and discharge. At TMAHD, patients had significantly higher TAC levels than at admission and discharge, both at 12:00 and 00:00 h. In conclusion, the absence of day/night serum TAC changes when clinically relapsed and the normalization of day/night serum TAC changes at TMHD can be considered as a biological marker of schizophrenia evolution.

## Introduction

Schizophrenia is a chronic mental disorder characterized by delusions, hallucinations, disorganized speech and behaviour, and other symptoms that cause social or occupational dysfunction [1]. This disease affects 0.5–1% of the worldwide population. Its biological aetiology is multifactorial and is still under investigation [2].

Free radicals (FRs) are compounds with unpaired electrons or an open shell configuration that may have positive, negative, or zero charge. Depending on the atom placed at its core, the radical can be described as oxygen, carbon, nitrogen or metal centred radical [3]. The unpaired electrons cause radicals to be highly reactive chemical molecules.

FRs have been involved in the pathophysiology of schizophrenia [4]. An excess of FR generation and/or an impairment of the antioxidant defence system (AODS) have been reported in schizophrenia [5]. FRs are partly responsible for negative symptoms [6], tardive dyskinesia [7], neurological soft signs [8] and cognitive impairment [9].

Increased, decreased or unchanged activity of antioxidant enzymes has been reported in patients with schizophrenia [10]. Blood concentrations of specific antioxidants can be measured individually, but those measurements are time consuming and expensive compared to the measure of the total antioxidant capacity (TAC) that reflects the antioxidant capacity of water-soluble molecules of plasma/serum. Synonymous of TAC are TAA (Total Antioxidant Activity), TAOP (Total Antioxidant Power), TAS (Total Antioxidant Status) and TAR (Total Antioxidant Response) [11].

Decreased [12–14] and unchanged [15] TAC concentrations have been reported in schizophrenic patients compared to healthy controls.

Several biological variables of the oxidant-antioxidant status such as, melatonin and malondialdehyde (MDA) present circadian and seasonal rhythms [16,17]. However, there is scanty information about TAC circadian rhythms in humans. A circadian rhythm of serum TAC levels, with higher levels at night (01:00 h) compared to daytime levels (13:00 h) [18] as well as higher midnight than midday levels [19] have also been reported in healthy subjects. In a recent study [13], it was published that acute relapsed paranoid schizophrenia patients present higher nocturnal than diurnal TAC levels when admitted to hospitalization, but three weeks later, at discharge, this difference was not present. There is no information about day/night differences in TAC levels of patients with schizophrenia in long-term studies.

The objective of this research is to study if there are day-night changes in serum TAC levels in a group of schizophrenia patients at admission, discharge and three month after hospital discharge (TMAHD).

## Materials and methods

Forty-eight subjects meeting DSM-IV diagnostic criteria for schizophrenia psychosis, paranoid type, comprised the initial sample of patients.

From the initial sample of 48 patients, at TMAHD, 35 patients were contacted by telephone; three of them did not attend to the control visit. Therefore, thirty-two subjects comprised the final study sample.

The patient’s clinical status was evaluated with the Clinical Global Impressions (CGI) scale [20]. Both, the severity of psychopathology (CGI-S) and the improvement (CGI-I) of the clinical evolution were evaluated by the same psychiatrist. To make antipsychotic treatments comparable, antipsychotic doses were transformed into chlorpromazine equivalent doses (CED) [21]. Haematological and general biochemical blood tests were carried out in order to exclude physical diseases.

The protocol study was carried out in accordance with the Helsinki declaration and was approved by the Ethic and Investigation Committee of the Canary Islands University Hospital. Written informed consent was obtained from all subjects after full explanation of the study.

TAC was measured to evaluate the antioxidant capacity. After 4 h of fasting, blood samples were collected at 12:00 (light period) and 00:00 (dark period) hours the day after admission, the day before discharge and at TMAHD. After each blood extraction, blood was placed in vacutainer tubes without anticoagulant. Blood was allowed to clot at room temperature during 15 minutes and then was centrifuged at 3000 rpm during 10 minutes. Serum samples were aliquot in Eppendorf tubes and kept frozen at -70° C until analysis.

Serum TAC was measured by the ABTS radical cation technique [22], with commercially available kits (Antioxidant Assay kit, SIGMA, Madrid, Spain). A detailed description of the technique has been published elsewhere. All serum samples were analysed the same day and by the same analyst, who was blind with respect to the characteristics of the samples.

Data were analysed with the 21st version of the Statistical Package for Social Sciences (SPSS, Illinois, Chicago, USA). The comparison of two quantitative variables was carried out by means of paired or independent t-test. The comparison of more than two quantitative variables was carried out with an analysis of variance (ANOVA) for repeated measures. If the result was significant, posterior multiple comparisons were carried out according to Bonferroni test. Chi-square was applied to analyse qualitative variable associations. All statistical tests were two-tailed and their significance level was set at 0.05. Quantitative data are presented as mean ± SD.

## Results

Table 1 shows that both samples, TMAHD sample (N = 32) and the only admission-discharge sample (N = 16), are comparable in the demographic and clinical data.

**Table 1:** Comparison of the demographic and clinical variables of both patients’ samples. Quantitative data are given as mean ± SD. Qualitative data are given as absolute frequency.

Variable	Admission-discharge	TMAHD	P
Age	35.6±9.5	37.8± 9.8	0.474
Gender (male/female)	12/4	23/9	0.371
Body Mass Index	27.1±5.6	27.3±5.2	0.902
Illness onset (years)	24.0±6.0	23.9±8.0	0.970
Illness duration (years)	11.3±8.8	12.9±10.9	0.666
NPA	3.6±2.9	4.3±3.5	0.608
Hospitalization days	21.9±14.3	21.1±7.5	0.864
CED (mgr./day)	799.2±420.9	729.3±392.2	0.634
CGI-S	3.1±0.86	3.0±1.0	0.800
CGI-I	2.0±1.0	1.8±0.86	0.541

TMAHD: Three Months After Hospital Discharge; NPM = Number of previous admissions; CED: Chlorpromazine equivalent dose; CGI-S: Clinical global impression, severity; CGI-I: Clinical global impression, improvement.

**Fig 1:**
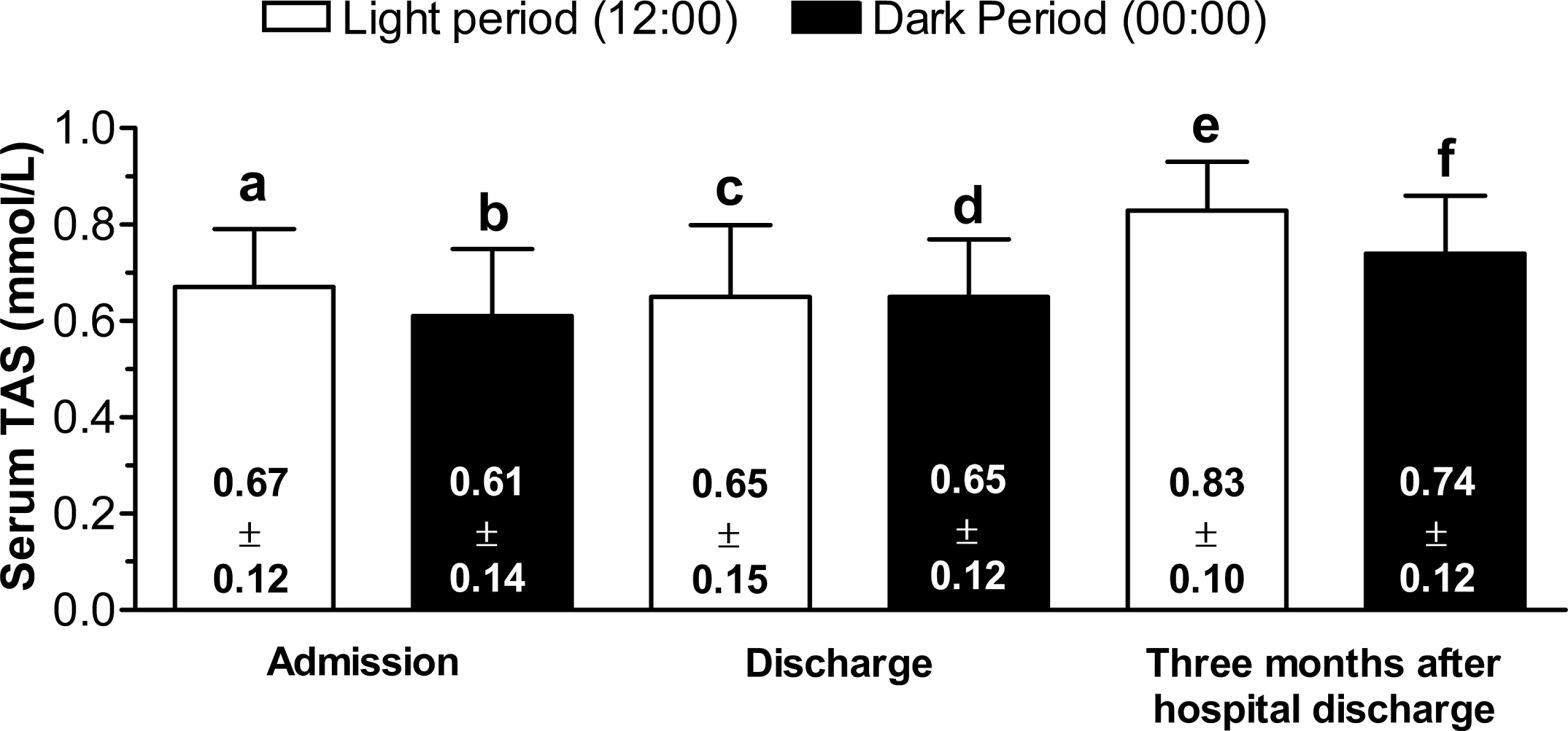
Bonferroni’s multiple comparisons of serum TAC levels. Day/night comparisons: a vs b, p: ns; c vs d, p: ns; e vs f, p < 0.006. Serum TAC levels comparisons of at the three time points in patients at midday and midnight: a vs c, p: ns; a vs e, p < 0.05; c vs e, p < 0.05; b vs d, p: ns; b vs f, p < 0.05; d vs f, p < 0.05.

The comparison of serum TAC levels in patients at midday and midnight at the three time-points was highly significant (midday: F = 14.9, p < 0.001; midnight: F = 7.7, p < 0.012). Multiple comparisons using Bonferroni’s correction showed that at admission and discharge patients had significantly lower serum TAC levels at 12:00 and 00:00 h than at TMAHD (p < 0.05). There were no significant differences between midday or midnight TAC levels at admission and discharge. Fig 1 shows the specific data.

If we consider the data of the control group published in a previous paper [13], the comparisons of serum TAC levels between patients and controls at midday and midnight showed that patients at 12:00 and 00:00 h had significantly lower serum TAC levels at admission and discharge than controls. At TMAHD, there were no significant differences in TAC levels between patients and controls neither at 12:00 nor 00:00 h. Table 2 shows the data.

**Table 2:** Comparison of TAC concentrations at 12:00 and 00:00 between patients and the control group.

Samples	TAS 12:00	P	TAS 00:00	P
Patients at admission	0.67±0.12	0.001	0.61±0.14	0.001
Control group	0.83±0.07		0.77±0.11	
Patients at discharge	0.65±0.15	0.001	0.65±0.12	0.001
Control group	0.83±0.07		0.77±0.11	
Patients at TMAHD	0.83±0.07	ns	0.74±0.12	ns
Control group	0.83±0.10		0.77±0.11	

TMAHD: Three Months After Hospital Discharge; ns: statistically not significant. Data are expressed as mean ± SD.

## Discussion

To the best of our knowledge, this is the first time that the absence at admission and discharge and then the existence of a day/night serum TAC concentration difference at TMAHD is reported in paranoid schizophrenia patients. This day/night difference was absent at admission and discharge despite the clinical improvement of the patients. However, at TMAHD, midday TAC concentrations were significantly higher than midnight concentrations. This pattern, higher at midday than midnight, has been reported in healthy subjects [19].

From our point of view, the absence at admission and discharge, and then the presence at TMAHD of a day/night TAC change suggests that patients have undergone a normalization of TAC levels. There are two facts that support this statement. First, the patients have recovered the pattern (higher at midday than at midnight) of TAC difference that is present in healthy subjects [13]. Second, the outpatient’s serum levels at midday and midnight are very similar to the serum levels of a group of healthy subjects [13].

We cannot know if this normalization is present in the serum of patients in the period between discharge and the check at TMAHD. Taking into account that the mean duration of the hospitalization period was 21.1 days (sd. 7.5), it is possible that the improvement in TAC figures would take more than one month because the change of serum TAC concentration between admission and discharge was not significant. A recent study [5] proposes that in schizophrenia patients low TAC levels can take until two and a half months to normalize.

As far as we know, this is the first time that TAC levels in schizophrenia patients at three different clinical points (admission, discharge, and at TMAHD) are compared. Serum TAC levels at TMAHD (00:00 and 12:00 h) are significantly higher than TAC levels at admission and discharge (00:00 and 12:00 h). There were no differences in TAC levels between admission and discharge at 00:00 or 12:00 h.

Some of the previous researches were carried out with a transversal design using inpatients [23] or outpatients [24,25] finding that patients had lower TAC levels than healthy subjects. Other studies applied a longitudinal design with outpatient being studied for a shorter period. Al-Chalabi et al. [26] studied the TAC level previous olanzapine treatment and two months later. They found that previous treatment; patients had lower TAC level than control subjects did. At two months of treatment olanzapine increased TAC levels compared to the basal level. Sarandol et al. [27] compared the TAC level of a combination of schizophrenia outpatients and inpatients before treatment and six weeks after treatment, finding that there was no change in TAC level.

In our opinion, the absence of difference between TAC levels at admission and discharge (12:00 and 00:00 h) is due to the relatively short period of time that exist between admission and discharge measurement (21.1 ± 7.5 days of hospitalization). The third TAC measurement was carried at TMAHD (approximately four times the admission-discharge period). The normalization of TAC levels is found at TMAHD, when serum TAC levels are similar between patients and healthy subjects. From the point of view of TAC levels, patients behave as healthy subjects at TMAHD.

There are several limitations in our study. First, the number of patient is limited. One of the disadvantages of studying circadian rhythms in humans, is that requires taking several biological measures [28] and because of the clinical characteristics of the patients (acutely relapsed), this is a difficult task to perform. Second, the effect of individual antipsychotics was not analysed because the number of subjects treated with each individual antipsychotic was small (less than five cases); this is the reason why we converted all antipsychotic treatment into CED. Our research also has some strengths. First, the same group of subjects comprises the sample of patients studied at admission, discharge and at TMAHD. Second, despite the small sample size, the patients improved significantly.

## Conclusion

It is very interesting to point the fact that not only the absolute values of TAC levels have normalized at TMAHD but also that the day/night rhythm that was absent at admission and discharge, appears at TMAHD. The present study adds more evidence that reduced TAC levels and the day/night changes may be involved in the pathophysiology of schizophrenia, although the specific role that these changes play in its aetiology remains unclear.
